# Concurrent measurement of nitrate and ammonium in water and soil samples using ion‐selective electrodes: Tackling sensitivity and precision issues

**DOI:** 10.1002/ansa.202000124

**Published:** 2020-12-05

**Authors:** Tolulope Fayose, Ellen Thomas, Tanja Radu, Peter Dillingham, Sami Ullah, Aleksandar Radu

**Affiliations:** ^1^ The Birchall Centre Lennard‐Jones Laboratories Keele University Keele Staffordshire UK; ^2^ School of Architecture Building and Civil Engineering Loughborough Leicestershire Loughborough University Leicestershire UK; ^3^ Department of Mathematics and Statistics University of Otago Dunedin New Zealand; ^4^ School of Geography Earth and Environmental Sciences and Birmingham Institute of Forest Research University of Birmingham Birmingham UK

**Keywords:** Bayesian statistics, ion‐selective electrodes, precision, sensitivity, soil analysis, standard addition

## Abstract

In this paper, we demonstrate the suitability, sensitivity, and precision of low‐cost and easy‐to‐use ion‐selective electrodes (ISEs) for concurrent detection of NH_4_^+^ and NO_3_^‐^ in soil and water by technical and non‐technical end‐users to enable efficient soil and water management exposed to chronic reactive nitrogen loading. We developed a simplified methodology for sample preparation followed by the demonstration of an analytical methodology resulting in improvements of sensitivity and precision of ISEs. Herein, we compared and contrasted ISEs with traditional laboratory‐based technique such as Flow Injection Analysis (FIA) and portable colorimetric assay followed by comparisons of linear regression and Bayesian nonlinear calibration approaches applied on both direct potentiometry and standard addition modes of analysis in terms of in‐field applications and improvement of sensitivity and precision. The ISEs were validated for sensing on a range of ambient soil and water samples representing a range of NH_4_^+^ and NO_3_^‐^ concentrations from pristine to excessive saturation conditions. Herein developed methodology showed excellent agreement with lab‐based and portable analytical techniques while demonstrating improvements in precision and sensitivity analysis illustrated by a decrease in confidence intervals by 50‐60%. We also demonstrated the utilization of the entire ISE response curve thus removing the biases originating from linear approximation which is often currently employed. Therefore, we show that ISEs are robust yet low cost and an easy to use technology that can enable high‐frequency measurement of mineral N and help to improve our understanding of N transformation processes as influenced by soil management, fertilization, land use, and climate change.

**Abbreviations**
ISEs: ion selective electrodes
FIA: flow injection analysis

## 1 INTRODUCTION

Of great concern is the increase in anthropogenic inputs of reactive nitrogen (Nr: oxidized and reduced forms of nitrogen) arising from the uses of nitrogen fertilizers, organic manures, sewage wastes, and fossil fuel. Their excessive use has more than doubled the input of Nr into terrestrial landscapes alone.^1^, ^2^, ^3^ While boosting crop production, excessive N fertilizer use in agriculture poses significant risks of losses of Nr into the air and water.^4^, ^5^, ^6^ Nr loss pathways in soils involve nitrate leaching, nitrate and ammonium run‐off into surface water, volatilization of ammonia, and emission of nitric oxide as well as nitrous oxide into the air– the latter being a potent greenhouse gas produced through denitrification and nitrification.^7^, ^8^, ^9^ Worryingly, the current global nitrogen use efficiency is only up to 45% by crops thus further enhancing Nr losses. Given that the main precursor Nr compounds are nitrate and ammonium and that it is these two species mainly that are mobilized from soils into water and air directly or through physicochemical and microbiological processes, it is imperative to devise management strategies for the reduction of losses of these species. While global to regional and local management strategies have been implemented for the reduction of Nr losses from the soil, the effectiveness of such strategies relies on accurate and spatiotemporally extensive monitoring of nitrate and ammonium concentration in soils and water to support timely reduction intervention actions.

The analysis of Nr in soil samples using traditional analytical techniques such as colorimetry, spectroscopy, and ion chromatography has become a standard practice in soil science.^10^, ^11^, ^12^, ^13^ However, it is worth noting the existence of some set‐backs; requirements of sample pretreatment from collection to extraction, long analysis time in the laboratories, deterioration of sample quality between collection and analysis (eg, microbial transformations and volatilization), and cost per sample analysis limits the suitability of nitrate and ammonia in environmental samples, particularly soils. Consequently, arable agricultural landscapes which are the hotspots of excessive Nr sources for loss into air and water are seldom tested (mostly once in 3 years) as a measure for planning fertilization. Thus from methodological, time, and cost perspectives of Nr monitoring, our ability to accurately measure real‐time and spatiotemporally extensive concentration of nitrate and ammonium in soils and water is a key impediment to achieving nutrient use efficiency.^14^, ^15^ This can often cause major barriers to the implementation of sustainable nutrient management in agriculture.

While sensors for monitoring in situ passive samples for quantifying Nr in soils and sediments have been developed (eg, ion exchange resin membranes and diffusive equilibrium in thin films‐DET^16^) these sensors still require post‐collection processing in the laboratory. A sensor for real‐time and in situ monitoring of nitrate and ammonium concentration in soils does not exist to our knowledge. There is an urgent need to develop a sensor that can measure nitrate and ammonium under field conditions at a cost and technical feasibility that can be operated by nontrained end users to help support the sustainable management of Nr in the environment and especially in the agricultural sector.

Development of a low‐cost and easy to operate nitrate and ammonium sensor would bring substantial environmental and economic benefits. The emerging new sensing technologies capable of addressing such requirements thus have to be very simple (in terms of construction and operation), and very cheap, while having sufficient analytical performance characteristics (sensitivity, selectivity, robustness, life time, etc.) to be able to monitor these nutrients at large. Ion‐selective electrodes (ISEs) have several advantages for application in environmental analysis; they can measure both bioavailable and extractable fraction of species of interest and are not affected by sample turbidity.^17^, ^18^ Their dynamic range that, under optimal conditions, routinely spans 6‐9 orders of magnitude^19^ is well suited for covering potentially wide variations of concentrations of target species. They can be miniaturized^20^ and easily integrated with mobile communication devices,^21^ and as such present an excellent opportunity for the development of a device for in situ sensing. Some reports indicate the possibility of their integration into large‐scale sensing networks^22^ opening an opportunity to address improvements in spatial and temporal measurement frequency as one of the key challenges for analytical chemists in environmental analysis.^23^ Indeed, recent reports on the utilization of ISEs for in‐field analysis of water^24^, ^25^, ^26^ and soils^15^, ^27^, ^28^ including even extraterrestrial soils^29^, ^30^ clearly indicate that ISEs are gaining ground in the field of environmental analysis.

However, despite the mentioned advances and opportunities, the confidence of practitioners to use ISEs is quite low illustrated by the very slow rate of their adoption for practical use in environmental analysis. Practitioners demand simple‐to‐use tools that will provide chemical information of sufficient quality to assist in the development of management decisions.^31^ The cost of development of relatively complex pre‐ and post‐analysis handling protocols required to address the complexity of samples (especially soils) and/or environmental conditions to obtain desired precision and sensitivity often outweigh the advantages of ISEs and put them in an inferior position in comparison to other instrumental techniques. Importantly, the development of modern analysis techniques is required to complement new technologies. Common current analysis practices for ISEs include unnecessary linear approximations that lead to bias and loss of information, poor specification of uncertainty for measurements, and no specification of uncertainty for important parameters such as slopes or limits of detection (LOD). This has previously motivated us to develop statistical methodologies such as non‐linear Bayesian calibration for improving precision^32^ and sensitivity^33^ of ISEs and tools^34^ that enable their use in practice by a non‐specialist.

The purpose of this paper is to demonstrate the capability of modern ISEs for accurate, precise, and simultaneous monitoring of the concentration of nitrate and ammonium in soils and water samples while maintaining the simplicity of operation. We approach this problem in two steps. Initially, we developed a simplified protocol for sample preparation and concurrent analysis NO_3_^‐^ and NH_4_^+^ in soils. This is validated against the traditional laboratory‐based flow injection analysis (FIA) and colorimetric techniques marketed for *in situ* analysis. We then proceed by applying Bayesian calibration methodology on direct potentiometry and standard addition mode of analysis in order to develop sensitive, precise, and fit‐for‐purpose ISEs. During this phase, we evaluate our methodology against traditional Nernstian, linear regression. The overall aim of this research was to create prototype sensors and protocols for nitrate and ammonium sensing that could potentially be adopted for application in the field for real‐time measurement of these ions in soil and water.

## 2 MATERIALS AND METHODS

### 2.1 Sensor preparation

#### 2.1.1 Reagents

Nonactin (ammonium ionophore I), sodium tetrakis[3,5‐bis‐ (trifluoromethyl)‐phenyl]borate (NaTFPB), tetradodecylammonium chloride (TDACl), tetrabutylammonium tetrabutylborate (TBA‐TBB) , high molecular weight heir poly(vinyl chloride) (PVC), bis(2‐ethylhexyl)‐ sebacate (DOS), 2‐nitrophenyl octyl ether (o‐NPOE), magnesium chloride (MgSO_4_), potassium nitrate (KNO_3_), ammonium chloride (NH_4_Cl), and tetrahydrofuran (THF) were obtained from Sigma‐Aldrich and of Selectophore grade. All aqueous solutions were prepared in ultra‐pure water obtained with Purelab Ultra water purification system (resistance 18 MΩ cm).

#### 2.1.2 Preparation of electrode substrate

Preparation of the sensing substrates was described in detail in our previous work,^13^ while schematic representation is illustrated in Figure S1. Briefly, for a single electrode, a 1.5 cm × 3.0 cm strip was cut from a parent acetate sheet and was subsequently etched with sandpaper (grit 240) for 30 s to provide the surface with enhanced surface roughness in order to allow for good adhesion of graphite. A simple graphite pencil (typically of high B value, eg, B4 or B6) was used to hand‐draw a line of carbon onto the roughened acetate strip. The conductivity of the line was checked by a simple multimeter until the resistance of ∼3 kΩ was achieved. Such acetate strip was then partially overlaid with a mask of non‐permeable sticky tape (eg, sellotape) on both sides. Importantly, a hole of 1 mm in diameter was punched on the tape used to cover the side with graphite and aligned with the graphite line as an aperture to allow for the application of ion‐selective membrane while the top end of the line was left uncovered in order to provide electrical contact.

For the preparation of sensing array, a larger strip was cut from the parent acetate sheet (capable to accommodate as many electrodes as desired) and an appropriate amount of lines were drawn. Note that sensing array included a polymer membrane‐based reference electrode that also required a graphite line. The procedure was then the same as for the preparation of a single electrode.

#### 2.1.3 Preparation of NO_3_^–^ and NH_4_^+^‐selective electrodes and reference electrode

The NO_3_^–^selective membrane contained 5.0 mmol/kg of TDACl, PVC (33.2 wt%), and *o*‐NPOE (66.4 wt %). NH_4_^+^‐selective membrane contained 10.0 mmol/kg of ammonium ionophore I and 5.0 mmol/kg of NaTFPB, PVC (32.9 wt%), and DOS (65.8 wt %). Reference electrode contained 10 mmol/kg of TBA‐TBB, PVC (33.2 wt %) and o‐NPOE (66.4 wt %). These represent the optimal membrane components we reported earlier in our previous study.^13^ All electrodes were prepared by dissolving the above‐mentioned components in 1.5 mL THF and the resulting cocktail was vortexed for 30 min for the complete dissolution of components.

For potentiometric measurements, an aliquot (∼20 μL) of relevant sensing membrane cocktail was drop cast onto the top of each electrode and left at room temperature to dry overnight. The following day, ISEs were conditioned in 1.0 x 10^‐3^ M of respective primary ion solution while reference electrodes were conditioned in 1.0 x 10^‐2^ M of KCl. In the case of sensing arrays, conditioning was performed in the solution containing 1.0 x 10^‐3^ M of NH_4_NO_3_ and 1.0 x 10^‐2^ M of KCl. All electrodes were conditioned for 18 h prior to the potentiometric experiments.

#### 2.1.4 EMF measurements

Potentiometric responses were recorded using a 16‐channel EMF‐16 interface (Lawson Labs Inc., PA) in a stirred solution. Initial evaluation of ISEs and reference electrodes measurements were performed against a double‐junction Ag/AgCl reference electrode with a 1 M LiOAc bridge electrolyte (Fluka). For measurement of analytes, a calibration step was initially carried out by immersing all electrodes into a beaker of appropriate background sample solution followed by stepwise addition of required standard solutions of NH_4_^+^ and NO_3_^‐^ using standard addition methods. Electrodes were properly rinsed with ultra‐pure water before immersing into the next sample to avoid carryovers. Potential responses (EMF) were then measured, and activities calculated from the calibration curve using the Nikolsky‐Eisenman equation.

#### 2.1.5 Selectivity measurements

For selectivity coefficient measurement, ammonium‐selective electrodes were prepared and conditioned overnight in 0.01 M NaCl, while nitrate‐selective electrodes were conditioned in 0.01 M (NH_4_)_2_SO_4_ overnight. Responses toward interfering ions were recorded according to the separate solution method as described by Bakker et al.^35^

### 2.2 Calculations and statistical analysis

Simulations and all nonlinear analyses were done using the OpenBUGS variant (version 3.0.3)^36^ of BUGS,^37^ linked to R‐studio^38^ using the R2WinBUGS library^39^ via the ISEtools library,^40^ and all other conversions or relationships for analysis are given in the supplementary section.

### 2.3 Analysis of natural water and soil samples

#### 2.3.1 Study sites and sampling

To investigate the performance of the sensor for practical application in soils, four sampling plots were selected randomly in four major land‐use types from around the North Wales and Staffordshire regions of the UK. Soil type 1 is a grassland denoted as GL; soil type 2 is improved grassland denoted as IGL; soil type 3 is an arable land denoted as AL, and soil type 4 comprises of forest soil. Samples of the latter were obtained from the Free‐Air Carbon dioxide Enrichment (FACE) facility in Staffordshire, the UK set up by The Birmingham Institute of Forest Research (BIFoR). They include samples around the three main tree species at BIFoR (ash, oak, and Scottish pine; denoted as ASH, OAK, and SP, respectively). Prior to sampling, overlying vegetation cover was removed and four soil cores (2‐15 cm depth; 5 cm diameter) were collected for each sample plot using a hand auger. The soil cores were collected from the corners of 1 × 1 m square on the chosen sample site, homogenized by manual mixing, and stored in gas permeable polyethylene bags before laboratory analysis. All samples, one travel blank and two filtered blanks were transferred on ice to the laboratory within 2 h of collection, where they were refrigerated at <5°C until needed for the experimental procedures. Immediately prior to use, all individual soil samples were sieved to 4 mm to remove plant materials, large stones, and earthworms followed by thorough mixing. Additionally, upstream and downstream water samples (n = 4) draining the BIFoR woodlands were sampled according to standard water sampling procedure.^41^, ^42^ Water analyzed for Nr included filtered and unfiltered samples since measurement using FIA required samples to be free of suspension. Pictures of sample location site are enclosed in Figure SI2.

#### 2.3.2 Background soil analysis

The main physicochemical soil analysis was performed on field moist soils, and according to established methods.^11^, ^27^ Soil moisture content was measured gravimetrically as moisture lost from a subsample of air‐dried soils by continuous heating (105^o^C) for up to 24 h until a constant weight was achieved. Soil pH was measured at (soil: water mix = 1: 2.5) by a standard pH probe. For all analysis, samples were blank corrected and precision was calculated. Results of background soil analysis are presented in Table SI1.

#### 2.3.3 Extraction procedure of NH_4_^+^ and NO_3_^‐^ from soil

A standard methodology for extraction of Nr requires a solution of 2 M KCl. We have also evaluated the potential of using 0.1 M MgSO_4_ as a single extracting medium for the simultaneous extraction of NH_4_^+^ and NO_3_^‐^. In all cases, 20 g of air‐dried sieved (< 2 mm) soil was weighed into 250‐mL HDPE Nalgene bottles. This was followed by the extraction of Nr from soil samples using 100 ml of the chosen solution as explained earlier.^43^ Briefly, the soil slurries (a combination of soil sample and extractant) were continuously shaken on a reciprocating shaker at 200 rpm for 1 h before being centrifuged at 4000 rpm for 30 minutes followed by a two‐step filtration into 20 mL scintillation vials through a no. 42 Whatman filter paper, and then 0.45 micron syringe filters (Whatman). All analysis was carried out immediately unless otherwise stated where samples were frozen until analysis.

#### 2.3.4 Determination of N_r_ in soil and water samples by FIA

The analysis of NH_4_^+^ and NO_3_^‐^ in soil type and water samples was performed on an automated Lachat flow injection analyzer (FIA) (Hach, Colorado, USA) according to standard colorimetric techniques.^11^, ^44^ Nitrate was measured by the cadmium reduction procedure, while ammonium was measured according to the Berthelot reaction. The limit of detection for NO_3_^‐^ was 0.03 mg N/L and for NH_4_^+^ 0.01 mg N/L. High extract samples were further diluted to obtain concentration within the calibration range of the instrument. The samples were blank corrected.

#### 2.3.5 Determination of N_r_ in soil and water samples using portable colorimetric assays

In addition to FIA, the sample concentrations of NO_3_^‐^ and NH_4_^+^ were validated using portable colorimetric assays. For NH_4_^+^ detection, the commercial LCK 303 (HACH LANGE GMBH, Germany) was used as follows; the cap zip of the commercial tube was unscrewed and carefully removed the foil from the screwed‐on cap zip. Then, 0.2 mL of sample was pipetted into the tube and the cap was immediately screwed back by fluting at the top. After that, the tube was shaken two to three times and left at room temperature for 15 min. Finally, the outside of the tube was cleaned with paper and placed into the reader. The method offered linearity in the range of 2.5‐60.0 mg/L. For NO_3_^‐^ detection, Palintest photometer 7100 (PHOT.23. AUTO) was used. Briefly, the Nitratest tube was filled until 20.0 mL mark. One leveled spoon of Nitratest powder and one Nitratest tablet was added and the tube was shaken for 1 min and left for 5 min or longer to ensure complete settlement of powders and to obtain a clear solution. The latter was carefully decanted into a round test tube and filled to 10.0 mL mark of tube. One Nitricol tablet was crushed and dissolved in 10.0 mL of clear solution. The tube was left for 10.0 min for the color to fully develop. Finally, the tube was placed into the detector. The method allowed linearity over a range of 0‐20 mg/L of NO_3_^‐^.

#### 2.3.6 Analysis of relevant background anions and cations in soil using ion chromatography (Dionex) and ICP‐AES, respectively

A 5 mL soil sample extract prepared as described in Section 2.1.2 was used without further dilution. The sample was injected directly into the ion chromatography instrument ((Dionex ICS2500, USA). Results are presented in Table S1.

For analysis of cations by ICP AES soil samples (0.5 g dry weight) were digested using 20 mL of conc. HNO_3_ in a microwave digestion. After digestion, samples were centrifuged and 1 mL of the supernatant solution was diluted by 10 mL (or 100 mL where necessary) to 1% HNO_3_. Diluted samples were analysed using ICP‐AES. Recalculated concentrations (to account for used dilution factor) are presented in Table S2.

## 3 RESULTS AND DISCUSSION

The work presented in this paper builds on our previous efforts to prepare simple and low‐cost ISE arrays.^13^ Such system allows simultaneous and concurrent analysis of NH_4_^+^ and NO_3_^‐^ while offering the benefits of the application of Bayesian non‐linear calibration methodology whose advantage is discussed below. Responses and analytical characterization of an ISE array used in this work are presented in Figure S4 and associated discussion. It is noteworthy that the limit of detections (LODs) obtained according to the classical IUPAC definition for ISEs were estimated as 5.3 × 10^‐6^ M (0.09 ppm) for NH_4_^+^ and 3.1 × 10^‐6^ M (0.2 ppm) for NO_3_^‐^. While this is not a correct definition for an LOD,^33^ it is useful as an initial estimate of the range where ISEs may be usefully employed.

### 3.1 Development of simplified sample preparation methodology

Plant nutrients in the soil have to be mobilized (extracted from the soil) in order to be analyzed. Furthermore, samples must be filtered in order to extract non‐dissolved particulate matter that can affect the operation of the analytical instrument. An important advantage of ISEs over classical instrumental techniques for soil analysis is that they are not affected by sample turbidity. This is perhaps the best illustrated by successful applications of ISEs in clinical analysis of whole blood. However, according to widely accepted practice NH_4_^+^ and NO_3_^‐^ are extracted from soils using 2 M KCl solution.^45^ The presence of such a high concentration of K^+^ and Cl^‐^ ions can significantly affect the response of ISEs and thus requires the identification of a suitable extraction solution.

#### 3.1.1 Suitability of extraction solution

Due to complexity of soils (potential large variations of ions and their concentrations both spatially and temporally) and the extent of the influence of interfering ions on response of ISEs, the choice of extraction solutions must be carefully examined. We present detailed analysis and discussion on suitability of extraction solution based on analytical performance of ISEs in the Supporting Information section Extraction solution. Based on our analysis, we conclude that the artificial addition of high concentration of K^+^ and Cl^‐^ (as 2 M KCl extraction solution) has a detrimental effect on the use of NH_4_^+^ ‐ and NO_3_^‐^ ‐ selective electrodes in soil analysis and suggest the use of 0.1 M MgSO_4_ as a suitable alternative.

#### 3.1.2 The efficiency of 0.1 M MgSO_4_ as a single extractant for analysis of Nr

**FIGURE 1:**
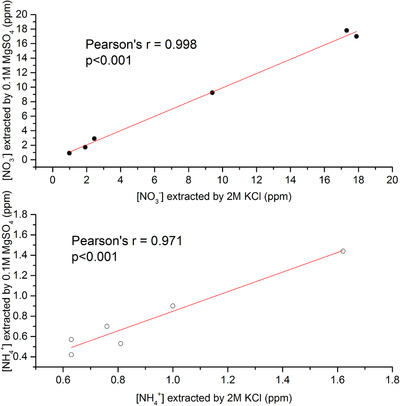
Comparison of the average concentration of extractable NO_3_^‐^ (top) and NH_4_^+^ (bottom) in soil and water samples obtained by extraction using 0.1 M MgSO_4_ and 2 M KCl. Results are obtained using FIA. Inset shows *r* and *P‐*value from the regression analysis for each ion

Analysis of common interfering ions in soil was performed by ion chromatography and ICP‐AES for anions and cations respectively. Results from Tables SI1 and SI2 show average concentrations of Mg^2+^ and SO_4_^2‐^ (9.2 and 2.0 mg/ L) found in the soils tested. This means that even after extraction with 0.1 M MgSO_4_, the resulting total concentration of each ion in the soil/extractant mixture still allowed for accurate measurement of NH_4_^+^ and NO_3_^‐^ without interference. To investigate the extraction efficiency of 0.1 M MgSO_4_, we compared the amount of extractable ions by 0.1 M MgSO_4_ with a standard extractant (2 M KCl). The values of NO_3_^‐^ and NH_4_^+^ determined by FIA when using the two investigated extractant solutions are presented in the Supplemental Info Table SI5. Figure 1 shows the correlation of the data.

A very good correlation between concentrations of soil nitrate (Pearson *r* = 0.998) and ammonium (Pearson *r* = 0.971) extracted with KCl and MgSO_4_ measured using standard laboratory instrumentation (FIA) method suggests adequate extraction efficiency of MgSO_4_ (98 ± 3 % and 96 ± 11 % for NO_3_^‐^ and NH_4_^+^ respectively). This confirms the possibility of using 0.1 M MgSO_4_ as an alternative for commonly used extractant. Further confirmation of minimal influence of 0.1 M MgSO_4_ as exctractant is presented in Figure SI4 and concomitant discussion. Therefore, 0.1 M MgSO_4_ was used in all subsequent analyses.

#### 3.1.3 Determination of Nr in water and soil samples using ISEs

In order to demonstrate the utility of herein described ISEs in the environmental analysis, we have collected a range of water and soil samples. In choosing soil samples, we rationalized that it would be important to demonstrate the utility of ISEs on all major land types. Due to the variety of factors, we have focused on the geographical location of North Wales and Staffordshire regions of the UK and we have identified four major land use types from around these regions. These were grassland (GL), improved grassland (IGL), arable land (AL), and forest soils. The latter were obtained from Birmingham Institute of Forest Research (BIFoR) and included locations around ash (ASH), oak (OAK), and Scottish pine (SP) trees. Note that BIFoR is an open‐air laboratory that focuses on understanding how the forest will respond to future increases in atmospheric CO_2_. It was our intention to demonstrate the utility of ISEs in the determination of Nr in forest soils and thus offer an additional tool for elucidation of potential change in biogeochemistry in our imminent future. In addition to soils, we have analyzed Nr in upstream and downstream water samples draining the BIFoR woodlands.

Determination of [NH_4_^+^] and [NO_3_^‐^] by ISEs was done using direct potentiometry method on filtered extracts as required for use by FIA. Measurements were performed against polymer membrane‐based reference electrode. Evaluation of its signal stability is illustrated in SI Figure SI6. Note that K^+^ is posing significant interference on the measurements of NH_4_^+^ if nonactin‐based ISEs are used as ammonium‐selective electrodes. Based on the background soil analysis (Table S3) and discussion in SI section ‘Influence of the naturally present [K^+^] on the determination of [NH_4_^+^]’ we conclude that ammonium‐selective electrodes used in this work are suitable for further application. Even though ISEs do not suffer from sample turbidity we have attempted measurement in samples with reduced pre‐treatment handling. Using four soil core samples around Scottish Pine, we have prepared a slurry containing 10% wt of soil in 0.1 M MgSO_4_. The slurry was stirred for ∼30 min prior to immersion of ISEs. There was no substantive difference between results obtained in such turbid samples and the ones obtained using traditional extraction and filtering (data not shown). Thus, this leads to a shortcut procedure for soil sample preparation and efficient use of ISEs in soil analysis with significantly reduced pretreatment handling.

**FIGURE 2:**
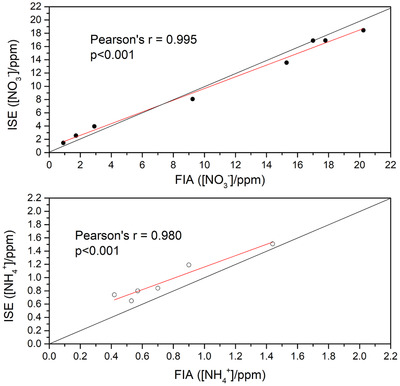
Comparison of the average concentration of extractable NO_3_^‐^ (top) and NH_4_^+^ (bottom) in soil and water samples obtained by ISE and standard method (FIA). Inset shows *r* and *P‐*value from the regression analysis for each ion

Initial analysis of samples using ISEs was performed using a classical linear approximation within the Nernstian region for illustrative purposes; later we compared it to a more sophisticated analysis approach using non‐linear Bayesian calibration. Results obtained using ISEs and verified against FIA are presented in Table S6. Figure 2 demonstrates the correlation of results obtained using the two techniques.

The Pearson coefficients (0.995 and 0.980 for NO_3_ and NH_4_^+^, respectively) show an excellent correlation between the two techniques. However, instead of prematurely concluding that ISEs can successfully substitute FIA in soil analysis, it is important to critically analyze results obtained for NH_4_^+^ as they excellently illustrate several issues that potentially lead to the rejection of ISEs as a tool for environmental analysis.

The ISE response is characterized by the logarithmic response to the activity of the target ion and a relatively large non‐linear section. As a consequence, a significant portion of the signal above noise levels is often neglected. In order to maintain brevity and focus, this somewhat unusual practice originating from the bias created by the current IUPAC definition and treatment of LOD (LOD_1969_) is discussed in the Supplemental Info (section “Bias in the determination of unknown activity around LOD of ISEs”) and elsewhere.^33^ This bias is nicely visible in Figure 2 bottom since the concentration of NH_4_^+^ in almost all samples is between LOD_1969_ and the limit of quantification (LOQ; as discussed in SI). Estimated LOD_1969_ and LOQ are 0.09 ppm and 0.9 ppm respectively. Please note that concentrations of NH_4_^+^ in water samples are not shown in Figure 2 due to their proximity to LOD_1969_, as they would normally be excluded in the classical Nernstian analysis approach.

### 3.2 Improving the precision and sensitivity of ISEs

Recently, we analysed the current IUPAC definition of LOD of ISEs and recommended a new LOD definition for ISEs that would be in line with broader IUPAC recommendations for a LOD.^33^ For practitioners, it is important that our recommendations realistic estimates of uncertainty. For brevity, we limited the demonstration of its utility on analysis of NH_4_^+^ in each soil core sample collected from BIFoR (the total of 12 samples) using four ISEs at a time.

In our analysis, we consider two analytical methodologies typically used in practice; direct potentiometry and standard addition. Briefly, the former is analogous to typical pH measurements, while the latter is highly recommended in cases where the sample matrix contributes to the analytical signal, for example, soil extracts.

Furthermore, the good analytical practice requires the treatment of drifts that is known to be a serious source of error.^46^ Drift can be especially pronounced in complex sample matrixes and as a consequence measurement protocols require cleaning steps and regular re‐calibration. Bayesian calibration addresses uncertainties of E^o^, slope, and selectivity ( $E^{0}$, $\frac{2.303RT}{z_{I}F}$, and $\sum a_{J}K_{I,J}^{pot}$ respectively from equation SI1), thus addressing all measurable contributions to random drift.^47^ Moreover, it combines results from all deployed electrodes while automatically weighting them based on individual precision.^48^ For example, noisier electrodes with poor slopes would have a lesser influence on estimates than less noisy electrodes with better slopes, yet still contribute information resulting in narrower credible intervals (the Bayesian analogy to confidence intervals). However, the model can still yield misleading results in the event of systematic drift unless further techniques are employed, e.g. standard addition to combat drift in E^0^. More details on the Bayesian calibration in the context of ISEs are provided in the Supplemental Info, section titled ‘Bayesian calibration in the context of ISEs’

**FIGURE 3:**
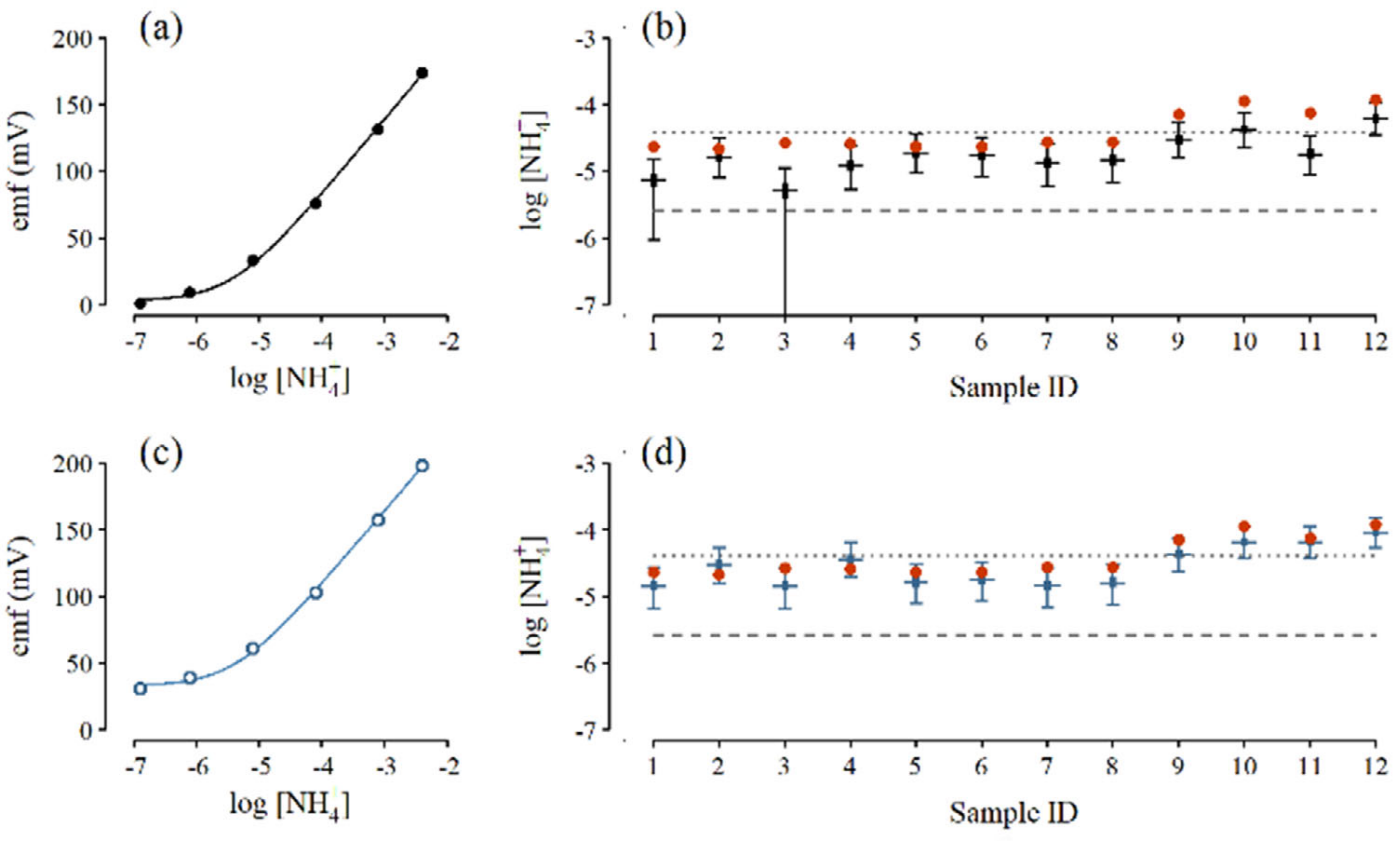
[NH_4_^+^] in soils samples obtained by direct potentiometry using single ISEs produce estimates different from each other ((A, B) ISE#1, black; (C, D) ISE#2, blue) and FIA (B, D, red circles). Calibration data (A, C, circles) and estimated calibration curves (A, C, lines) are combined with sample emf responses to estimate [NH_4_^+^] (B, D). Error bars in (B, D) indicate 95% credible intervals; middle 50% indicated by thick bars; wide dashes (going through the middle of the bars) represent Bayesian point estimates using the posterior median. Also shown are the traditional LOQs (B, D dotted line at 0.7 ppm = 4.1 ×10^‐5^ M) and the Bayesian LOD estimate (B, D, dashed line at 0.05 ppm = 2.6 ×10^‐6^ M)

**FIGURE 4:**
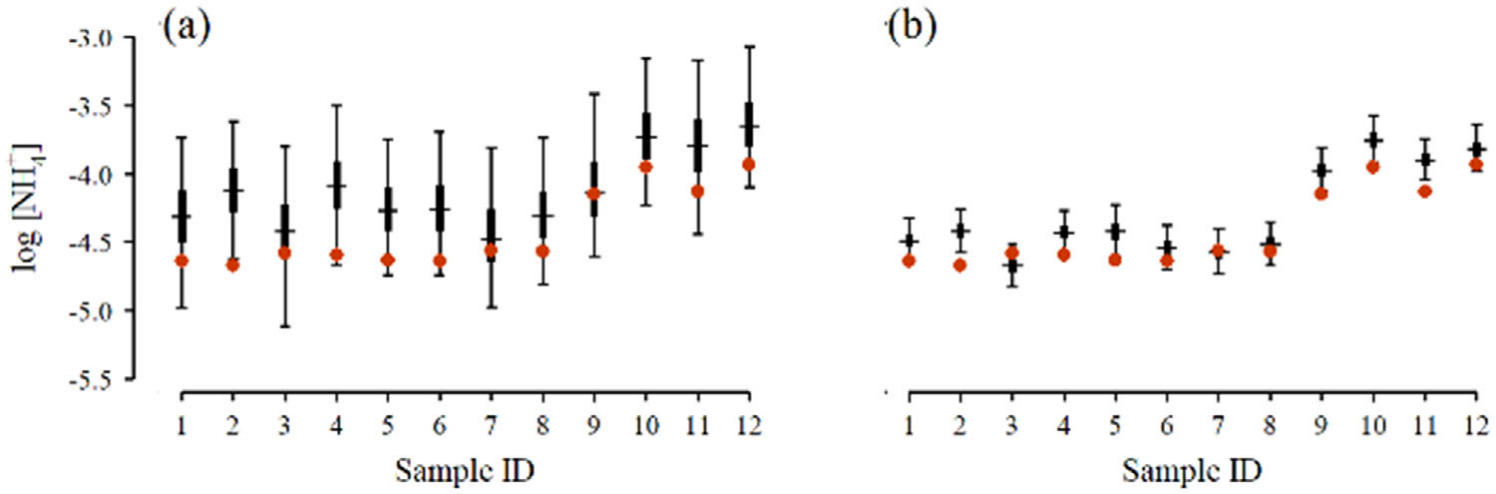
[NH_4_^+^] in soils samples obtained by direct potentiometry (A) and the standard addition method (B) using an array of four ISEs; [NH_4_^+^] measured by FIA are overlayed (red circles). Error bars indicate 95% credible intervals; middle 50% indicated by thick bars; wide dashes represent Bayesian point estimates using the posterior median

Figures 3 and 4 illustrate the significance of treatments of drifts and the utilization of multiple ISEs in both direct potentiometry and standard addition mode of measurements. Figure 3 shows the results of the analysis of NH_4_^+^ in 12 soil samples using two different single ISEs in direct potentiometry mode with their associated calibration curves. Results are compared against the results with the ones obtained with FIA (red circles). The error bars represent a measure of precision, while mean deviation from FIA values allows an assessment of accuracy. For ISE#1, calibration data fit the theoretical model well (Figure 3A) but consistently underestimated [NH_4_^+^] relative to FIA (Figure 3B), often by half an order of magnitude and struggling to detect values well above its nominal LOD (Figure 3B, Samples 1, 3 versus dashed line). In contrast, ISE#2 was more consistent with FIA results (Figure 3D). Further, by using the non‐linear calibration curve, ISE#2 was able to achieve reasonable accuracy and precision below the traditional definition of LOQ (Figure 3D, Samples 1‐8 versus dotted line). The poor accuracy observed for ISE#1 is often associated with baseline drift in the ISE combined with direct potentiometry. Due to the harsh environment of soil extracts, analysis of 12 samples might have required re‐calibration mid analysis, or use of standard addition methods that are impervious to baseline drift (though not to systematic drift in other parameters).

Figure 4 demonstrates the difference in precision and accuracy between direct potentiometry and standard addition methods when Bayesian calibration is applied to an array of four ISEs. In both cases, there is good agreement between ISEs and FIA. However, mean absolute residuals (log scale) using standard addition mode are reduced by over 50% (0.29 versus 0.14), demonstrating improved accuracy of standard addition. Similarly, the mean credible interval width is 3.5 times larger for direct potentiometry versus standard addition, likely due to inconsistent estimates from individual ISEs caused by drift, demonstrating the improved precision from standard addition. That is, in settings where non‐negligible drift is likely to occur, standard addition would be expected to produce clearly superior estimates than direct potentiometry. Therefore, standard addition method must be the preferred mode of analysis. It does not require frequent re‐calibration and addresses matrix effect while the application of multiple ISEs alongside Bayesian calibration significantly improves the precision and sensitivity of measurements. As a result, the entire calibration curve is utilized thus eliminating the need for the use of artificially set limits of quantification while confidence intervals are reduced by about 50‐60% in comparison to a single ISE.

For readers interested in adopting Bayesian calibration and estimation for ISEs, an R package, ISEtools, is available.^40^ ISEtools has an extensive help file with examples; there is also a shorter introduction available via open access.^34^ The current version of ISEtools assumes units of mol/L and operates on the log_10_ scale. Because all calculations are done on quantiles from the underlying distribution, estimates and intervals from ISEtools can be simply converted to ppm without creating bias, e.g. $x_{\mathrm{ppm}}=10^{x_{\mathrm{molar}}}\times \mathrm{molecular}\;\mathrm{mass}\times 1000$. Future versions of ISEtools will accommodate ppm directly.

## 4 CONCLUSIONS

Herein we demonstrated that the multisensor array allowed concurrent determination of NH_4_^+^, and NO_3_^‐^ thus leading to drastically simplified handling protocols. We have shown that 0.1 M MgSO_4_ can effectively substitute the 2 M KCl as an extraction solution while there is no need for filtration of the extract. Furthermore, We measured NH_4_^+^, and NO_3_^‐^ in a range of soil and water samples and evaluated analytical data against standard laboratory technique (FIA). Excellent correlation (Pearson's *r* = 0.980 and *r* = 0.995 for NH_4_^+^, and NO_3_^‐^ respectively) indicated the potential for use of ISEs in environmental analysis. Moreover, we compared and contrasted ISEs versus colorimetric assay in terms of portability and applications in situ and concluded that utilization of multi‐electrode assays of modern ISEs can be superior to current portable techniques. Importantly, we demonstrated that non‐linear Bayesian calibration significantly improves the precision and sensitivity of measurements. As a result, the entire calibration curve is utilized thus eliminating the need for the use of artificially set LOQ while confidence intervals are reduced by about 50‐60% in comparison to single ISE. Our analysis is applied to two standard analytical practices; direct potentiometry and standard addition. We demonstrated that the standard addition method must be the preferred mode of analysis. It does not require frequent re‐calibration and addresses matrix effect while the application of multiple ISEs alongside Bayesian calibration significantly improves the precision and sensitivity of measurements.

This work demonstrates that modern ISEs are a powerful tool for mineral N analysis in soil and water, especially when considering the demand for a significant increase in the frequency of analysis with reduced per‐sample and per‐measurement costs. High frequency measurement of mineral N can help improve our understanding of the impacts of land use, soil and climate change on N transformation processes together with losses of reactive N from soil into air and water.

## CONFLICT OF INTEREST

The authors declare no conflict of interest.

## Supporting information

Supporting Information
