# Supplementary material for: Concurrent measurement of nitrate and ammonium in water and soil samples using ion‐selective electrodes: Tackling sensitivity and precision issues
Source: Anal Sci Adv. 2020 Dec 5;2(5-6):279–88. doi: 10.1002/ansa.202000124 (PMC10989628; doi:10.1002/ansa.202000124)
Supplement: Supplementary file 1 — Supporting Information [file ANSA-2-279-s001.docx]

Supplemental Information

Concurrent measurement of nitrate and ammonium in water and soil samples using ion-selective electrodes: tackling sensitivity and precision issues

Tolulope Fayose^1^, Ellen Thomas^1^, Tanja Radu^2^, Peter Dillingham^3^, Sami Ullah^4*^, and Aleksandar Radu^1*^

^1^ The Birchall Centre, Lennard-Jones Laboratories, Keele University, Keele, Staffordshire, ST5 5BG, United Kingdom

^2^ School of Architecture, Building and Civil Engineering, Loughborough University, Loughborough Leicestershire, LE113TU, United Kingdom

^3^ Department of Mathematics and Statistics, University of Otago, Dunedin 9054, New Zealand

^4^ School of Geography, Earth and Environmental Sciences, and Birmingham Institute of Forest Research, University of Birmingham, Birmingham, B15 2TT, United Kingdom

(Key words; reactive nitrogen, soil analysis, ion-selective electrodes, nutrient sensing, Bayesian modelling.)

ABSTRACT: In this paper, we demonstrate the suitability, sensitivity and precision of low-cost and easy-to-use ion-selective elec-trodes (ISEs) for concurrent detection of NH4+ and NO3- in soil and water by technical and non-technical end users to enable efficient soil and water management exposed to chronic reactive nitrogen loading. We developed a simplified methodology for sample preparation followed by the demonstration of an analytical methodology resulting in improve-ments of sensitivity and precision of ISEs. Herein we compared and contrasted ISEs with traditional lab-based technique such as Flow Injection Analysis (FIA) and portable colorimetric assay followed by comparisons of linear regression and Bayesian non-linear calibration approaches applied on both direct potentiometry and standard addition modes of analysis in terms of in field applications and improvement of sensitivity and precision. The ISEs were validated for sensing on a range of ambient soil and water samples representing a range of NH_4_^+^ and NO_3_^-^ concentrations from pristine to excessive saturation conditions. Herein developed methodology showed excellent agreement with lab-based and portable analyti-cal techniques while demonstrating improvements in precision and sensitivity analysis illustrated by a decrease in confi-dence intervals by 50-60 %. We also demonstrated the utilization of the entire ISE response curve thus removing the bi-ases originating from linear approximation which is often currently employed. Therefore, we show that ISEs are robust yet low cost and an easy to use technology that can enable high frequency measurement of mineral N and help improve our understanding of N transformation processes as influenced by soil management, fertilization, land use and climate change.

Contents

[Experimental 4](#_Toc57800031)

[Figure SI1: Schematic representation of pencil-drawn ion selective electrode 4](#_Toc57800034)

[Figure SI2. Pictures of soil sampling sites 5](#_Toc57800035)

[Figure SI3. Pictures of sampling sites at BIFoR 6](#_Toc57800036)

[Table SI1. Background soil analysis for three types of soil sampled around North Wales and Staffordshire. Four replicates sampled for each soil type. 6](#_Toc57800037)

[Table SI2. Analysis of soil anions by ion chromatography 7](#_Toc57800038)

[Table SI3. Elemental analysis of soil cations by ICP-AES 7](#_Toc57800039)

[Discussion 7](#_Toc57800040)

[Analytical characterization of an ISE array 7](#_Toc57800041)

[Figure SI4. Responses of NO3- - and NH4+ - selective electrodes in the background of deionized water 8](#_Toc57800042)

[Portable colourimetric assay vs ISEs 8](#_Toc57800043)

[Extraction Solution 10](#_Toc57800044)

[Table SI4. Selectivity coefficients and experimental slopes for selected ions obtained for fabricated NO_3_^-^ - and NH_4_^+^ - selective electrodes using a separate solution method and a minimum of 4 electrodes. 11](#_Toc57800045)

[The efficiency of 0.1 M MgSO4 as a single extractant for analysis of Nr 12](#_Toc57800046)

[Table SI5. Analysis of soil ammonium and nitrate extracted by 2 M KCl and 0.1 M MgSO4 using flow injection analyzer (FIA). 12](#_Toc57800047)

[Figure SI5. Response curves of NO3- electrode (top) and NH4+ electrode and (bottom) in different background samples 15](#_Toc57800048)

[Table SI6. Bioavailable NH4+ and NO3- in water and soil obtained using FIA (4 replicate measurements) and ISEs (4 different electrodes, Nernstian approximation). 15](#_Toc57800049)

[Influence of naturally present [K^+^] on the determination of [NH_4_^+^] 16](#_Toc57800050)

[Evaluation of polymer membrane-based reference electrode 16](#_Toc57800051)

[Figure SI6. Evaluation of signal stability of TBA-TBB-based reference electrode 17](#_Toc57800052)

[Bayesian calibration in the context of ISEs 17](#_Toc57800053)

[Figure SI7. Relationship between data, priors, posteriors, and final estimates of sample activity 18](#_Toc57800054)

[Bias in the determination of unknown activity around LOD of ISEs 18](#_Toc57800055)

[Figure SI8: Illustrations of bias in the determination of the unknown activity relative to the different definitions of LOD 20](#_Toc57800056)

[Equations for conversion 20](#_Toc57800057)

[Literature 21](#_Toc57800058)

# Experimental

Fully prepared electrode with membrane (gray)

Acetate sheet

etched with sand paper

Conductor sealed with sellotape with punched hole to allow dropcasting a membrane

Conductor line drawn

by pencil type 3B

## Figure SI1: Schematic representation of pencil-drawn ion selective electrode


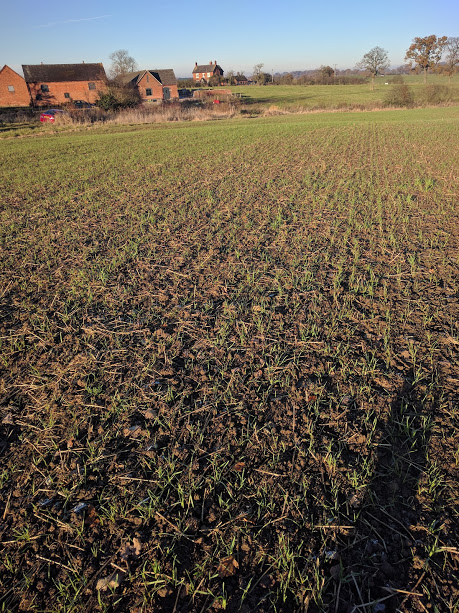

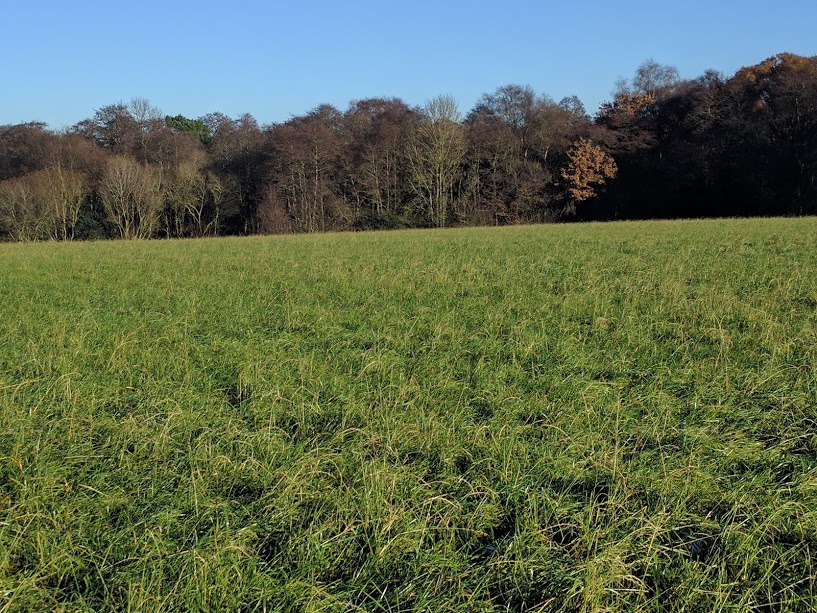

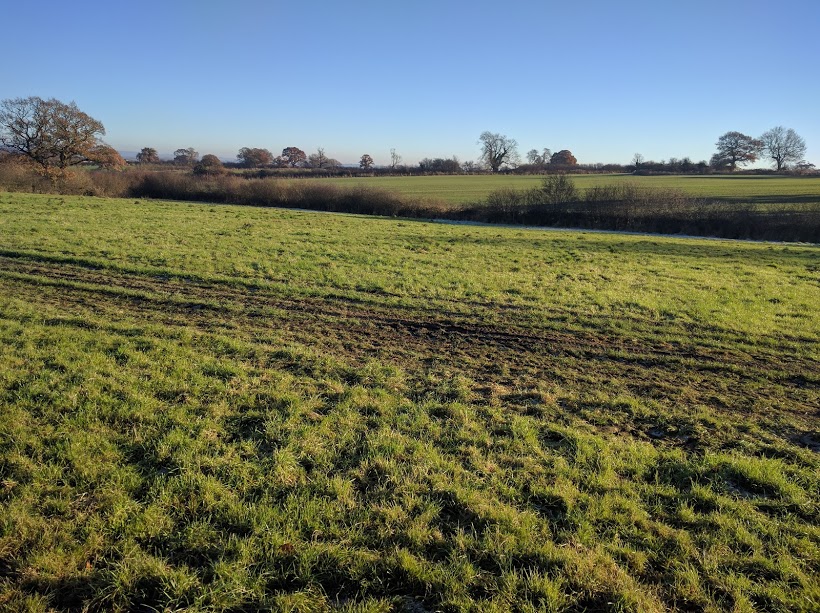


Figure SI2. Pictures of soil sampling sites. Top left) grassland (GL); top right) improved grassland (IGL); bottom left) Arable land (AL).


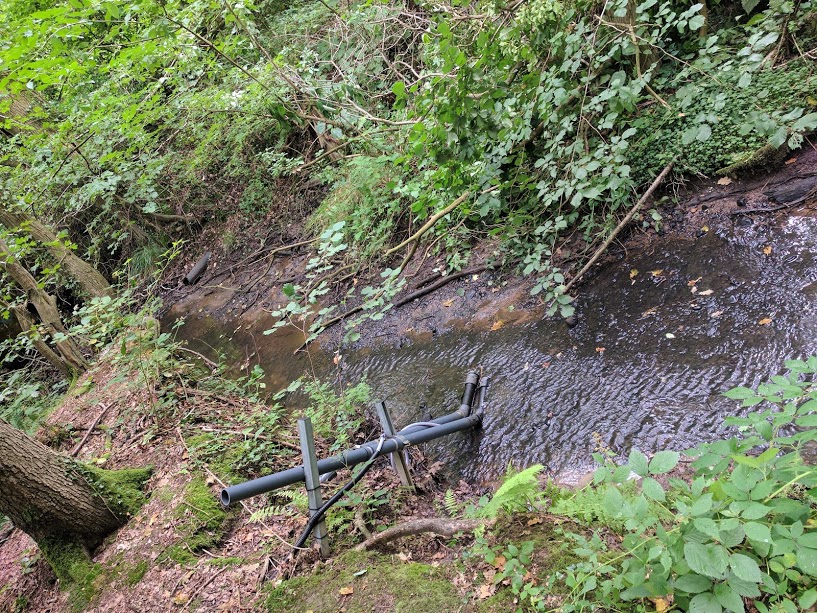

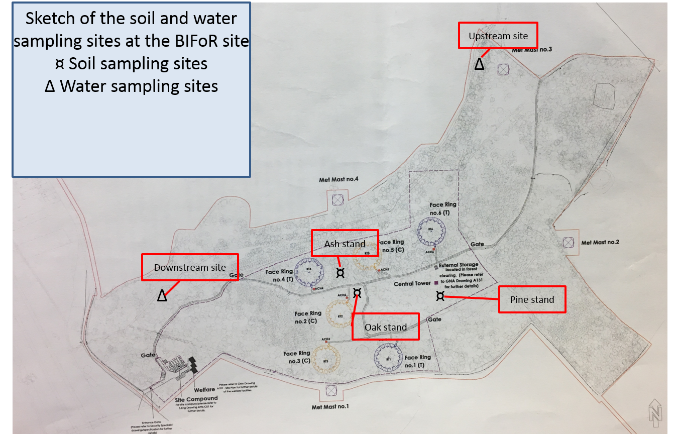


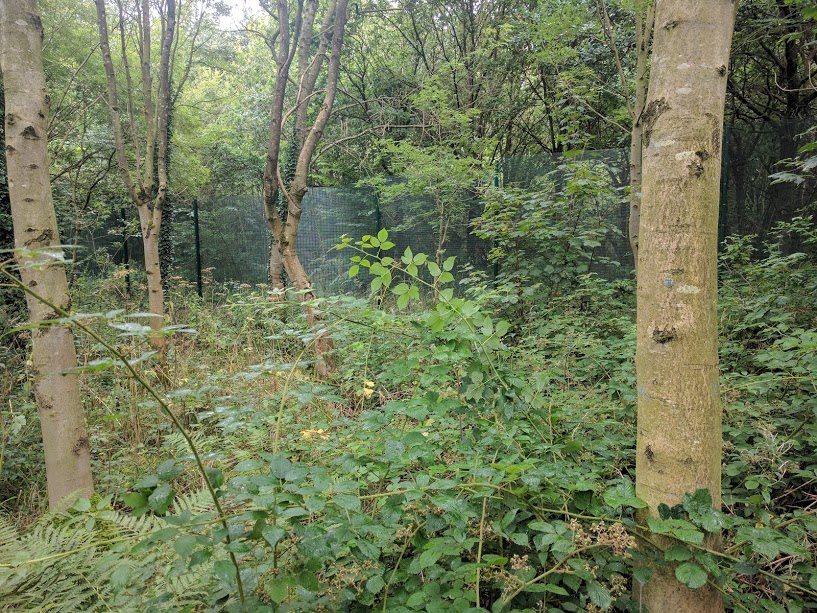

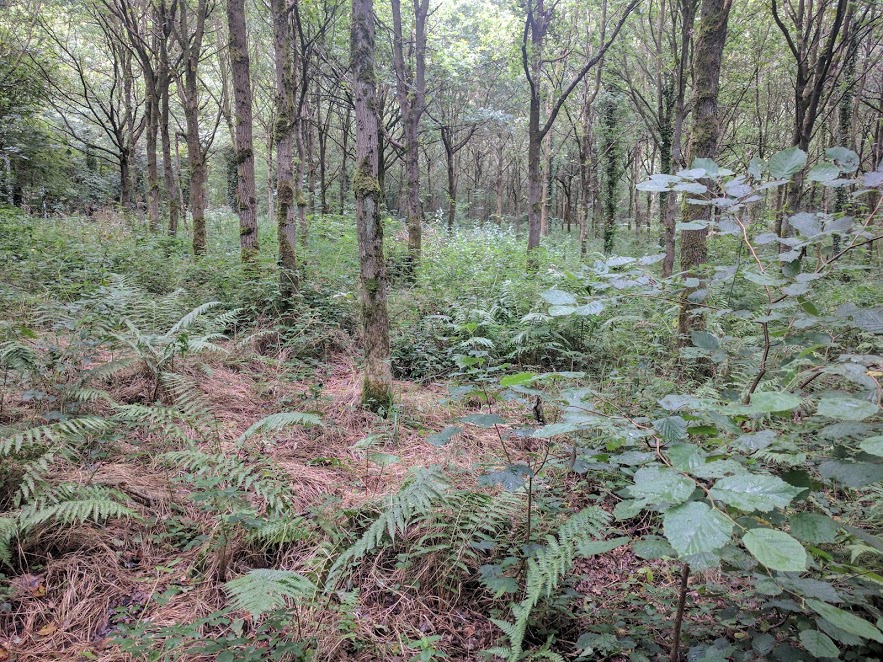


Figure SI3. Pictures of sampling sites at BIFoR. Top left) Map of BIFoR annotated with the location of sampling sites. Top right) photograph of a water sampling site. Bottom) Photographs at forest sampling sites.

## Table SI1. Background soil analysis for three types of soil sampled around North Wales and Staffordshire. Four replicates sampled for each soil type.

| Soil sample | pH | Moisture content |
| --- | --- | --- |
|  |  | (g/ g) |
| GL | 6.66 ± 0.03 | 0.14 ± 0.05 |
| IGL | 6.26 ± 0.03 | 0.08 ± 0.02 |
| AL | 6.38 ± 0.02 | 0.15 ± 0.03 |
| ASH | 6.14 ± 0.04 | 0.35 ± 0.04 |
| OAK | 5.86 ± 0.02 | 0.29 ± 0.05 |
| SP | 6.03 ± 0.02 | 0.14 ± 0.03 |

## Table SI2. Analysis of soil anions by ion chromatography

|  | Sample Concentration in (mg/ L ± SD) | | | | | | |
| --- | --- | --- | --- | --- | --- | --- | --- |
| Samples | F^-^ | Cl^-^ | B^-^ | NO_2_^-^ | NO_3_^-^ | H_2_PO_4_^-^ | SO_4_^2-^ |
| GL | 0.4 ± 0.2 | 0.9 ± 0.3 | NA | NA | 16 ± 2 | 2.2 ± 0.7 | 3 ± 1 |
| IGL | 0.12 ± 0.02 | 0.9 ± 0.6 | NA | NA | 16 ± 4 | 7 ± 1 | 2.1 ± 0.8 |
| AR | 0.63 ± 0.07 | 2.0 ± 0.3 | NA | NA | 8 ± 2 | 0.7 ± 0.3 | 2.0 ± 0.4 |
| Ash | 0.13 ± 0.05 | 0.9 ± 0.4 | NA | NA | 0.77 ± 0.04 | NA | 0.7 ± 0.2 |
| Oak | 0.03 ± 0.04 | 0.8 ± 0.3 | NA | NA | 1.7 ± 0.6 | NA | 1.0 ± 0.3 |
| SP | 0.19 ± 0.06 | 5 ± 2 | NA | NA | 2.8 ± 0.8 | NA | 3.2 ± 0.7 |

## Table SI3. Elemental analysis of soil cations by ICP-AES

|  | Sample Concentration in (mg/ L ± SD) | | | |
| --- | --- | --- | --- | --- |
| Sample Labels | K | Na | Ca | Mg |
| GL | 1.9 ± 0.4 | 3.2 ± 0.7 | 21 ± 2 | 3.1 ± 0.8 |
| IGL | 2.8 ± 0.8 | 4 ± 1 | 47 ± 3 | 6.1 ± 0.7 |
| AR | 1.0 ± 0.1 | 1.5 ± 0.5 | 17 ± 2 | 2.6 ± 0.5 |
| Ash | 0.5 ± 0.3 | 1.1 ± 0.4 | 5.6 ± 0.6 | 15 ± 3 |
| Oak | 0.3 ± 0.4 | 0.9 ± 0.1 | 6.4 ± 0.4 | 12 ± 2 |
| SP | 0.4 ± 0.1 | 1.4 ± 0.3 | 6.4 ± 0.1 | 16 ± 2 |

# Discussion

## Analytical characterization of an ISE array

Figure SI4 demonstrates simultaneous responses of NH_4_^+^ - and NO_3_^-^ - selective electrodes prepared as an array of 4 electrodes for each ion and measured against polymer membrane-based reference electrode. Please note that the suitability of using such reference electrode has also been evaluated in the past.^1^ For clarity reasons response of only one electrode for each ion has been shown; other electrodes responded similarly. Responses have been recorded in the background on deioninized water. Obtained slopes of (57.6 mV/decade for NH_4_^+^ and 55. 4 mV/decade for NO_3_^-^) were near-Nernstian and thus deemed as satisfactory for further use. Limit of detections (LODs) obtained according to the classical IUPAC definition for ISEs were estimated as 2.5 x 10^-6^ M (0.04 ppm) for NH_4_^+^ and 3.1 x 10^-6^ M (0.2 ppm) for NO_3_^-^. While this is not a correct definition for an LOD,^2^ it is useful as an initial estimate of the range where ISEs may be usefully employed.


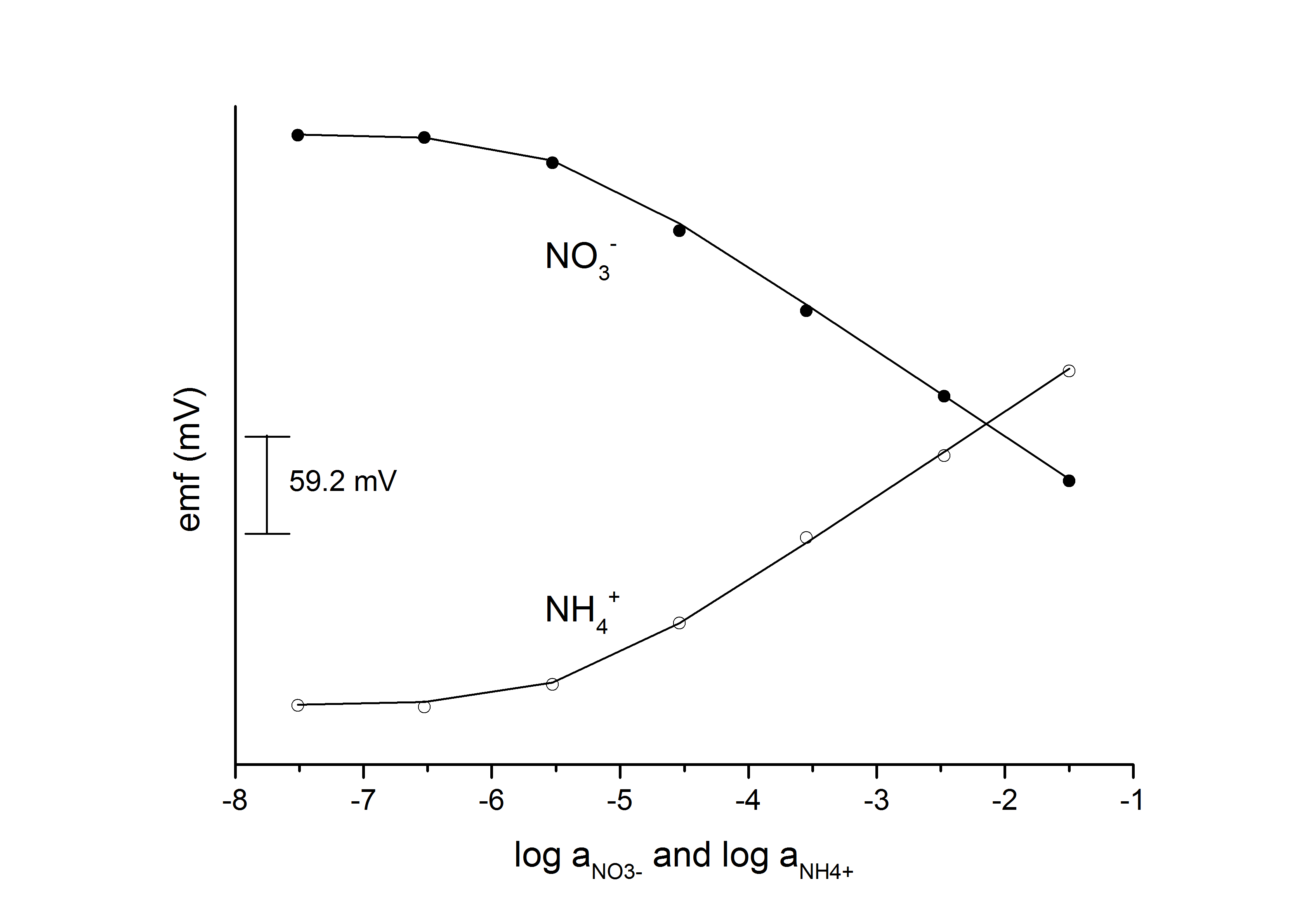


Figure SI4. Responses of NO3- - and NH4+ - selective electrodes in the background of deionized water (full circles and open circles respectively) and measured against TBA-TBB-based reference electrode as characterized in Fayose et al.

## Portable colourimetric assay vs ISEs

|  | **Colourimetric assay** | **ISEs** |
| --- | --- | --- |
| **Instrumentation** | Colourimeters with built-in programmes for analysis of a variety of ions are readily available such as the two we used in this work. To reduce the cost, a tech-savvy person can use online tutorials to prepare simple colourimeters but would have to develop their methodology using existing reagents. | Voltammeter with input impedance that is typically ~1000 times larger than the resistance of an electrode.^3^ Handheld data loggers for ISEs are readily available with the need to buy ISEs separately. A tech-savvy person can use numerous online tutorials to make a simple potentiometer.^4^ We have also demonstrated the preparation of wireless multichannel sensing system.^5^ |
| **Sample preparation** | *Very involved* | *Minimal* |
|  | As per section 2.12 in the main body of the paper, NH_4_^+^ and NO_3_^-^ have to be first extracted from soil sample typically by shaking the sample in the presence of 1M KCl. This is followed by two-step filtering; initially through typical qualitative lab filter for removing bigger particles followed by 0.45 microns syringe filters. This standard lab practice does not require highly specialized space and personnel, and albeit time consuming in principle can be done at home. However, soil samples would need to be collected before fertilization campaign. Depending on the size of the farm (average farm size in the UK is 77 ha although 47% of all holdings are up to 20 ha)^6^, type of crops, season, type of fertilizer, to name just a few major factors, it is easy to imagine that required number of samples can easily be in double digits if not more. It is easy to envision that such an analysis could require more time, energy, and perhaps even a dedicated person. | While there is still a need for ion extraction, filtering is not necessary as discussed in the main paper body. The slurry prepared by appropriate amounts of soil and water is stirred followed by immersion of NH_4_^+^- and NO_3_^-^-selective ISEs. On the contrary to colourimetry, ISEs do not suffer from sample turbidity. There were no significant differences between results obtained between ISEs in slurry and filtered samples (data not shown). |
| **Calibration** | *Nonexistent* | *Simple* |
|  | Typically built-in in the commercially available kits. | Direct Potentiometry mode: In analogy to pH meters, at least two point calibration is necessary **prior** the analysis.  Standard addition mode: No prior handling needed. Upon extraction of soils, electrodes are immersed in the extract followed by the addition of at least aliquot of a standard solution into the extract. |
| **Sample analysis** | *Very involved* | *Relatively simple* |
|  | It can be seen from section 2.12 in the main body of the paper that analysis of NH_4_^+^ involves a waiting step of 15 min, while analysis of NO_3_^-^ is even more complex as involves two steps of mixing required chemicals with the sample and waiting for a pre-specified time. While this may not be problematic in the analysis of one sample, the length of analysis and involvement of personnel in the analysis of multiple samples increases significantly. For example, in this work, a fully trained PhD student took ~4 h to complete analysis of 64 samples (32 samples for each of NO_3_^-^ and NH_4_^+^ ions) in a fully equipped lab. Therefore, it is easy to imagine that analysis can take an additional day of work of at least one person. | Direct Potentiometry mode: In analogy to pH meters, sample analysis is done by dipping of an electrode into the sample solution and collecting the reading.  Standard addition mode: Performed in conjuction with calibration. The unknown concentration is determined using the difference in signal readings for sample and sample with the added standard. |
| **Precision and efficiency** | Calculation of concentration of an analyte in typically done using Lamber-Beer law. Built-in programmes typically specify a concentration range where linearity is observed. If the concentration in the sample falls in this range, accuracy and precision of analysis are sufficient for routine analysis of nutrient level. However, if the concentration of the sample is outside of the range, the analysis complexity rises, as re-analysis is necessary. Furthermore, colourimetric assays are by default single analyte analytical technique. Samples are analysed in a sequence of analytes. | The dynamic range of ISEs can be multiple orders of magnitude thus enabling the capture of wide concentration ranges. However, due to the logarithmic dependence of signal versus the concentration, the precision is affected with pronounced negative effects if the concentration of the sample falls in the curvilinear response range as discussed below. At present only single ISEs are commercially available. However, due to the drive towards miniaturization, robustness, and simplicity of ISEs, it is easy to envision the emergence of instrumentation containing multi-ISEs. This could be very important in the development of multi-ion ISE assays thus significantly simplifying analytical procedures, while simultaneously improving the precision of analysis as discussed in the paper. |
| **Data processing and data logging** | Depends on the sophistication of the instrument and the experience and skill of the analyst. Typical low-end instruments show the result on the screen and the user has to record it. Interestingly, the need to manually record data seem to be a major obstacle for farmers. According to one report, only 21% of farmers in the UK use the electronic form of record keeping.^7^ Therefore, increasing spatial and temporal frequency of analysis necessarily involves further manual work on creating and maintaining a database. While beneficial, the amount of needed work is coupled with daily challenges including financial investment, skill level and complexity of the technology, knowledge transfer, and even personal attitude^8^, often leads to complete refusal to adopt the technology. Recently, we are witnessing an influx of technologies that integrate sensing with software-based data processing, logging, and transmission. | |

## Extraction Solution

Table SI4 provides measured selectivity coefficients obtained for NH_4_^+^ - and NO_3_^-^ - selective electrodes against selected ions deemed the most likely to be present in the soil samples and capable of influencing the response of ISEs. It also provides slopes obtained while determining response to each interfering ion. Standard approaches to estimating selectivity coefficients assume Nernstian slopes, and the importance of satisfying this assumption was a topic of many works in the field of ISEs.^9^ Since all slopes are near-Nernstian, the estimated selectivity coefficients are of practical use.

## Table SI4. Selectivity coefficients and experimental slopes for selected ions obtained for fabricated NO_3_^-^ - and NH_4_^+^ - selective electrodes using a separate solution method and a minimum of 4 electrodes.

|  | NH_4_^+^ |  |  | NO_3_^-^ |  |
| --- | --- | --- | --- | --- | --- |
| Ion | $log K_{i, J}^{pot}$ ± S.E | Slope ± S.E | Ion | $log K_{i, J}^{pot}$ ± S.E | Slope ± S.E |
| Na^+^ | -2.96 ± 0.03 | 57.5 ± 0.3 | Cl^-^ | -2.48 ± 0.02 | -52.9 ± 0.3 |
| Cs^+^ | -2.56 ± 0.03 | 54.05 ± 0.06 | NO_2_^-^ | -1.27 ± 0.03 | -53.6 ± 0.5 |
| K^+^ | -0.98 ± 0.02 | 55.2 ± 0.2 | I^-^ | 1.4 ± 0.03 | -55.2 ± 0.1 |
| Ca^2+^ | -4.54 ±0.01 | 25.1 ± 0.2 | ClO_4_^-^ | 3.25 ± 0.02 | -54.3 ± 0.2 |
| Mg^2+^ | -4.33 ± 0.02 | 26.9 ± 0.1 | SO_4_^2-^ | -4.72± 0.09 | -25.2 ± 0.4 |
| NH_4_^+^ | 0 | 56.4 ± 0.3 | NO_3_^-^ | 0 | -55.8 ± 0.2 |

Table SI4 nicely illustrates the need for knowledge of selectivity coefficients. For example, positive values of selectivity coefficients for I^-^ and ClO_4_^-^ indicate that the electrode is more sensitive to these two ions then NO_3_^-^. Therefore their presence in samples may strongly influence the results of the analysis. However, practical importance is based on selectivity and the relative concentrations likely to be encountered. Fortunately, the concentration of I^-^ and ClO_4_^-^ in soils under ordinary conditions are typically low enough to actually pose no significant interference. Nevertheless, it is important to understand the conditions that can lead to the rise of concentration of strongly interfering ion(s) in order to evaluate the potential for use of ISEs for any specific application.

In the case of NH_4_^+^, potassium ion presents as the key interference. K^+^ is naturally present in soils at various concentrations depending on the soil type and use. Artificial addition of high concentration of K^+^ and Cl^-^ (as 2M KCl extraction solution) has a detrimental effect on the use of NH_4_^+^ - and NO_3_^-^ - selective electrodes in soil analysis. Using the value for selectivity coefficient and Nickolskii-Eisenmann’s equation it can be calculated that such high levels of K^+^ and Cl^-^ would lead to the increase of LODs to approximately 10^-2^ M (~140 ppm) and 3 x 10^-3^ M (~107 ppm) for NH_4_^+^ and NO_3_^-^ respectively. These LODs are unsuitable for the determination of NH_4_^+^ and NO_3_^-^ in natural samples, indicating that a different extraction solution is necessary.

From Table SI4, selectivity coefficients for Mg^2+^ and SO_4_^2-^ are the smallest indicating that they pose the least interference to NH_4_^+^ - and NO_3_^-^ - selective electrodes respectively. We therefore decided to evaluate 0.1 M of MgSO_4_ as extraction solution.

## The efficiency of 0.1 M MgSO4 as a single extractant for analysis of Nr

Table SI5. Analysis of soil ammonium and nitrate extracted by 2 M KCl and 0.1 M MgSO4 using flow injection analyzer (FIA). Ammonium was analyzed using the Berthelot reaction, and nitrate was analyzed using the automated cadmium-reduction method. Results are an average of four replicate samples

|  | NO_3_^-^ ± SE (mg/ L ) | | NH_4_^+^ ± SE (mg/ L) | |
| --- | --- | --- | --- | --- |
| Soil sample | 2 M KCl | 0.1 M MgSO_4_ | 2 M KCl | 0.1 M MgSO_4_ |
| GL | 18± 3 | 17 ± 2 | 0.8 ± 0.3 | 0.7 ± 0.2 |
| IGL | 17 ± 4 | 18 ± 2 | 1.0 ± 0.2 | 0.9 ± 0.2 |
| AL | 9 ± 2 | 9.2 ± 0.4 | 0.81 ± 0.08 | 0.53 ± 0.08 |
| ASH | 1 ± 2 | 1 ± 1 | 0.6 ± 0.5 | 0.4 ± 0.4 |
| OAK | 2 ± 1 | 2 ± 1 | 0.6 ± 0.6 | 0.6 ± 0.6 |
| SP | 2 ± 1 | 3 ± 1 | 1.6 ± 0.4 | 1 ± 0.4 |

Figure SI5 showing responses of NH_4_^+^ - and NO_3_^-^ - selective electrodes in deionized water and 0.1 M MgSO_4_ further confirms the minimal influence of the new extract solution of the response of electrodes.

In order to illustrate the strong influence of 2M KCl solution on the electrodes’ response Figure SI5 also contains electrode response in this solution modelled using the Nikolsky-Eisenman equation and experimentally available parameters.

In the background of 0.1 M MgSO_4_ near - Nernstian slopes of 54.25 mV/dec and -53.91 mV/dec and LODs of 5.0 x 10^-6^ M (0.09 ppm) and 4.0 x 10^-6^ M (0.25 ppm) were observed for NH_4_^+^ and NO_3_^-^ electrodes respectively. This is in agreement with previously studied ammonium and nitrate ISEs. ^10–14^


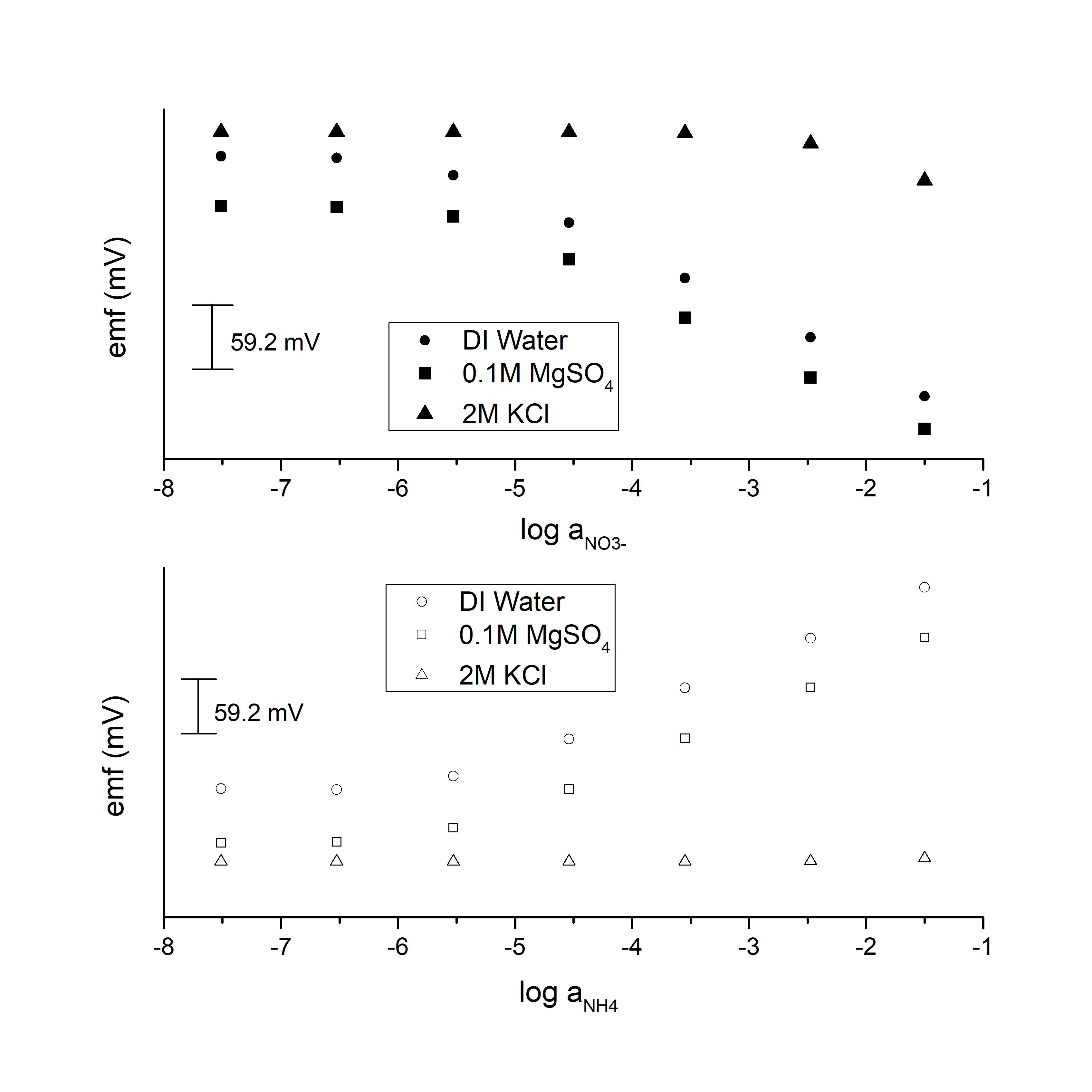


Figure SI5. Response curves of NO3- electrode (top) and NH4+ electrode and (bottom) in different background samples: circles represent solution sample of ultra-pure water, squares represent an initial background solution of 0.1 M MgSO_4_, and triangles represent calibration in 2 M KCl modelled by the Nikolsky-Eisenman equation:

$E=E^{0}+\frac{2.303RT}{z_{I}F}log(a_{I}+\sum a_{J}K_{I,J}^{pot}$) (1)

where a_I_, a_J_ and $K_{I,J}^{pot}$ symbolize ion of interest, interfering ion, and selectivity coefficient respectively. Symbols E, E^0^, R, T, z, and F have their usual meaning of potential, standard potential, gas constant, temperature, ion charge and Faraday constant.

Table SI6. Bioavailable NH4+ and NO3- in water and soil obtained using FIA (4 replicate measurements) and ISEs (4 different electrodes, Nernstian approximation). Presented values are averaged estimates with associated standard errors (SE).

|  | NO_3_^-^ ± SE (mg/ L ) | | NH_4_^+^ ± SE (mg/ L) | |
| --- | --- | --- | --- | --- |
| Soil sample | FIA | ISE | FIA | ISE |
| GL | 17 ± 2 | 17 ± 4 | 0.7 ± 0.2 | 0.8 ± 0.5 |
| IGL | 18 ± 2 | 17 ± 4 | 0.9 ± 0.2 | 1.2 ± 0.3 |
| AR | 9.2 ± 0.4 | 8 ± 2 | 0.53 ± 0.08 | 0.6 ± 0.1 |
| ASH | 1 ± 1 | 2 ± 2 | 0.4 ± 0.4 | 0.7 ± 0.6 |
| OAK | 2 ± 1 | 3 ± 3 | 0.6 ± 0.6 | 0.8 ± 0.3 |
| SP | 3 ± 1 | 4 ± 3 | 1 ± 0.4 | 1.6 ± 0.3 |
| DS | 15 ± 6 | 14 ± 6 | 0.28 ± 0.07 | 0.2 ± 0.3 |
| US | 20 ± 2 | 18 ± 1 | 0.33 ± 0.07 | 0.2 ± 0.2 |

## Influence of naturally present [K^+^] on the determination of [NH_4_^+^]

It is well known that nonactin-based ISEs used for the determination of NH_4_^+^ suffer from strong influence by present K^+^. The selectivity coefficient is determined as logK^pot^= -0.98±0.02 (Table SI4). In essence, thi smeans that if the [K^+^] in the sample is an order of magnitude higher than [NH_4_^+^] the ISE is not able to distinguish between these two ions. According to Table S3, the highest [K^+^] is in GL and IDL samples (1.85 x 10^-6^M and 2.82 x 10^-6^M respectively). Therefore, using eq 1 it could be calculated that [NH_4_^+^] at LOD will be 3.17 x 10^-7^M and 3.86 x 10^-7^M respectively. These values are bellow determined values of 7.0 x 10^-7^M 9.0 x 10^-7^M (Table SI6) and thus we are able to proceed with the analysis.

## Evaluation of polymer membrane-based reference electrode

Perfromance of TBA-TBB-based reference electrode was evaluated against Ag/AgCl electrode. Figure SI6 illustrates the protocol and the results obtained. Following the conditioning, the TBA-TBB reference electrode was immersed in 0.1M KCl and signal was monitored against Ag/AgCl along side additional Ag/AgCl electrode. The response of the later is presented in pink, while the response of the former is in blue. Electrodes were then subsequently immersed in series of solutions with varying ions and their concentrations. The stability of the signal encouradged us to use TBA-TBB-based electrode in subsequent measurements.


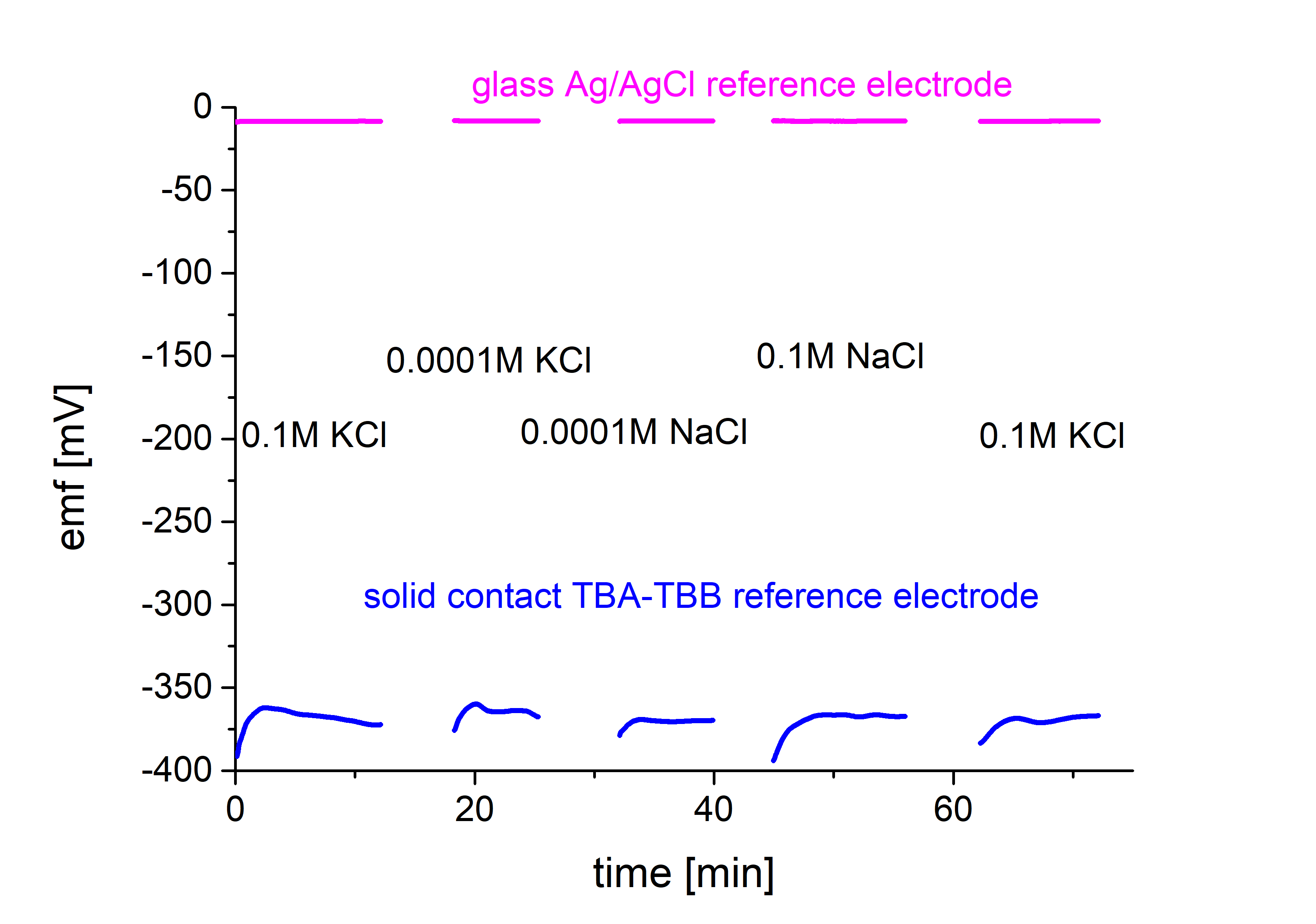


Figure SI6. Evaluation of signal stability of TBA-TBB-based reference electrode. Blue trace) signal of TBA-TBB-based reference electrode recorded against Ag/AgCl reference exposed to solutions of NaCl and KCl of different concentrations as illustrated in the figure. Pink tace) response of a control Ag/AgCl reference electrode recorded against Ag/AgCl reference.

## Bayesian calibration in the context of ISEs

Bayesian statistical analysis is based on the concept of prior knowledge or beliefs about random variables (Pr(A)) being combined with a model relating data to those variables (Pr(B|A) to form updated beliefs about the variables, given the collected data (Pr(A|B)). The updated beliefs are called the posterior probability distribution of A given B. In an ISE calibration context, A represents the unknown variables (analyte concentrations and calibration parameters) while B represents the data (all calibration data and sample emf values). Figure SI6 illustrates how the posterior calibration parameters are first estimated before the final estimation of analyte activities and calibration intervals. For more detailed explanation and model specification, please see Dillingham et al and associated Supplemental Info.^15^


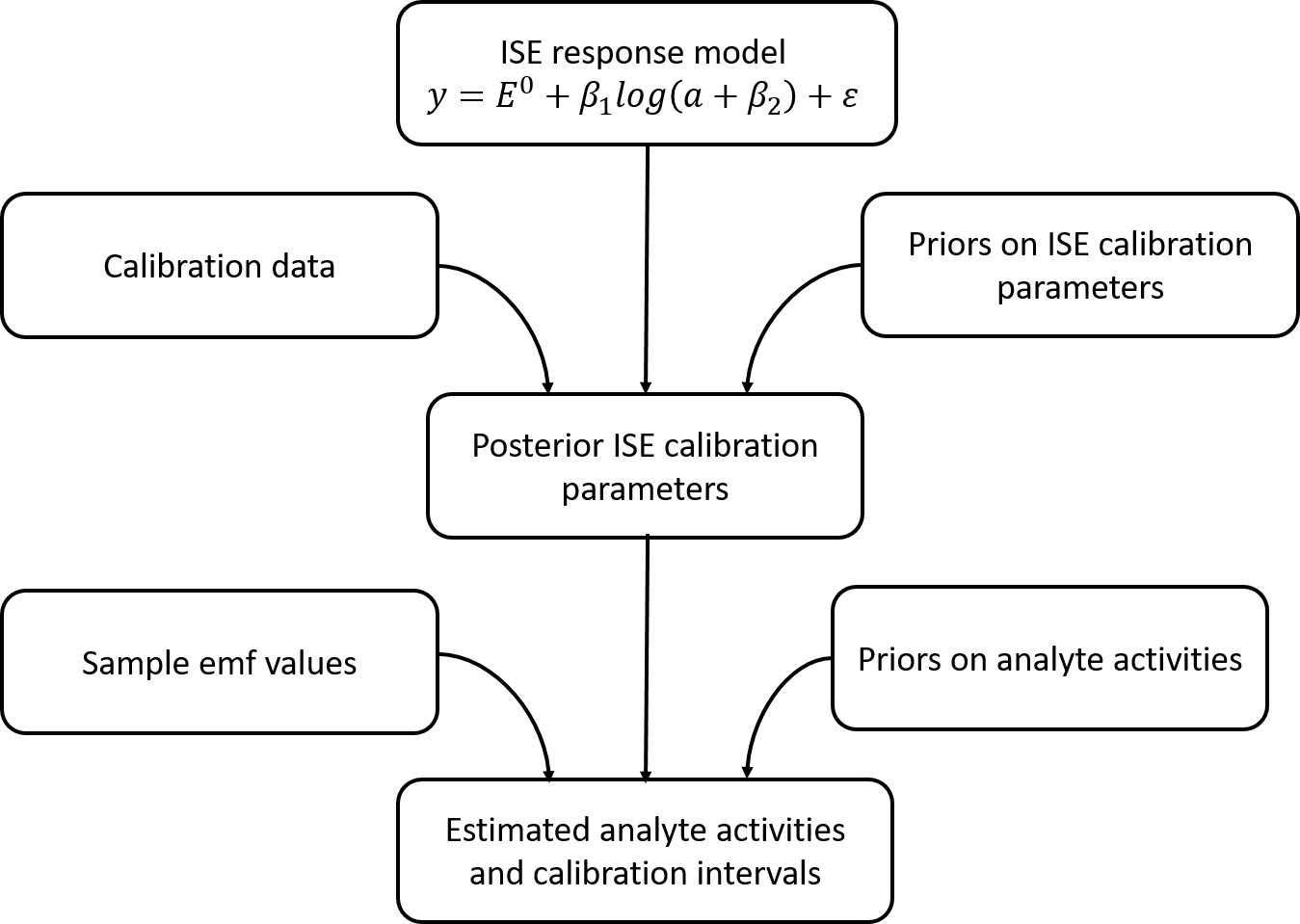


Figure SI7. Relationship between data, priors, posteriors, and final estimates of sample activity. In the model equation β_1_ is the slope parameter related to the temperature and the charge of the primary ion, a is the activity of the primary ion, and β_2_ relates to the selectivity and activity of interfering ions. Errors, ε, are assumed to follow a normal distribution with constant variance σ^2^.

## Bias in the determination of unknown activity around LOD of ISEs

Despite IUPAC broadly defines LOD as a signal-to-noise ratio the LOD of ISEs is defined in a somewhat unusual manner. Due to a relatively large non-linear response, the LOD is defined as the cross-section of the two straight lines representing situations of the complete absence of target ion (a_I_ = 0) and interfering ions (a_J_ = 0) presented as dotted lines in Figure SI7. According to Eq 1, responses below the LOD (a_I_=0) have a constant value and represent base line. When the activity of primary ions becomes sufficiently high, the term $\sum a_{J}K_{I,J}^{pot}$becomes negligible and the equation is transformed into Nernst Equation with the slope of 59.2/z_I_ mV/decade (at 25 ^o^C). Although such a definition has certain advantages, it does not meet IUPAC recommendations. This definition is introduced in practice in 1969^16^ so we refer to it as LOD_1969_. One of its key disadvantages is illustrated by the shaded area in Figure SI6. It represents the substantial bias created in the determination of unknown activity due to the projection into Nernstian response rather than using actual ISE response. As a result, practically useful Limit of Quantification (LOQ) is defined at about 1 order of magnitude above LOD_1969_.

Unfortunately, the current definition of LOD and the need for such high LOQ lead to neglecting a significant portion of the signal above noise levels. As it can be observed in Figure SI6, between the signal obtained in a typical signal-to-noise as S/N = 3 and LOQ, the neglected signal spans almost 2.5 orders of magnitude. This was acceptable in a clinical analysis where ISEs made the most significant scientific and commercial impact since the levels of blood electrolytes are typically 2-3 orders of magnitude above LOD_1969_. However, utilization of ISEs in environmental analysis with the view of simplification of sensing protocols and reducing the cost of analysis requires the utilization of the entire response curve. In other words, the luxury of neglecting usable signal above the noise levels is not acceptable for in situ deployed devices.


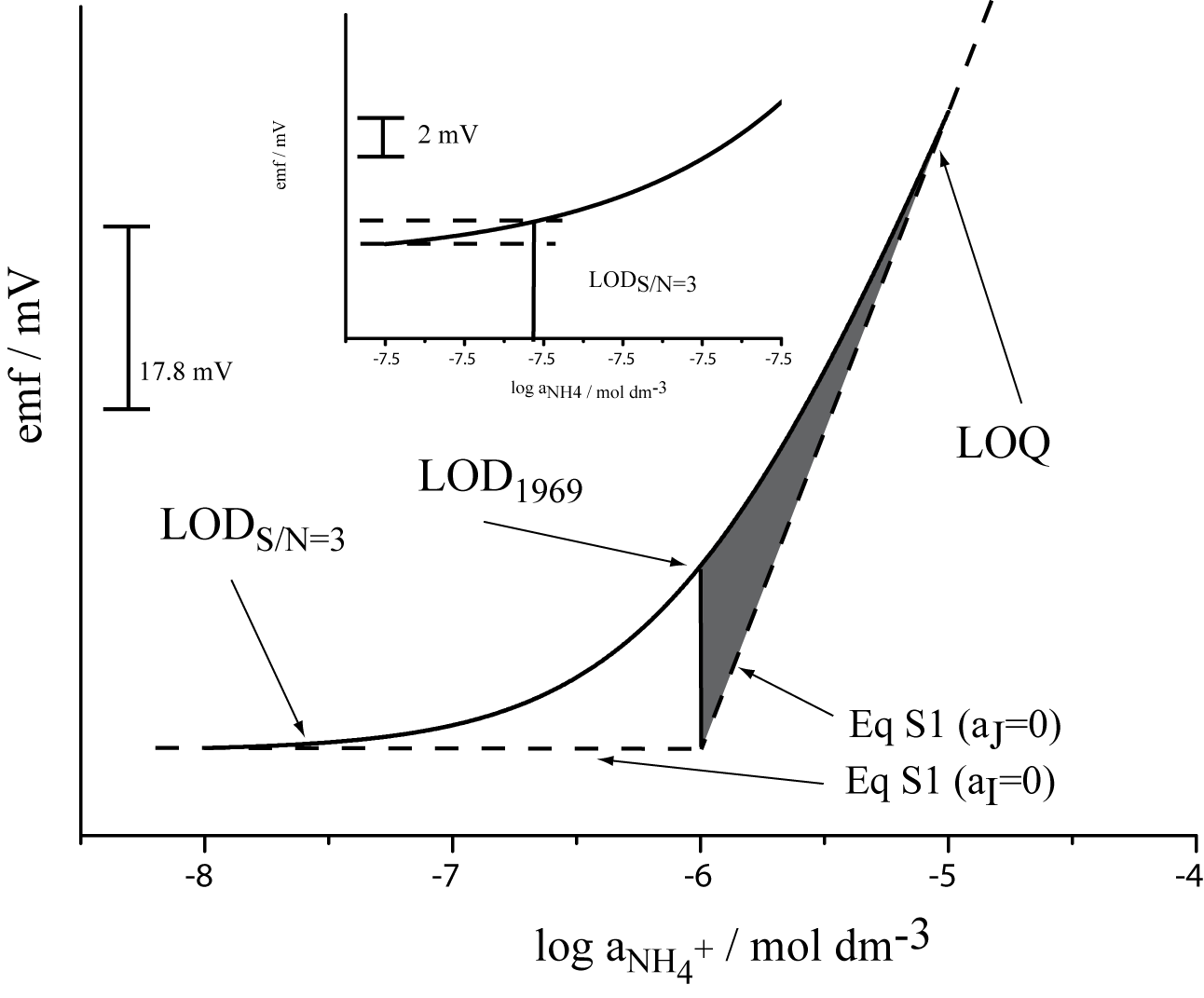


Figure SI8: Illustrations of bias in the determination of the unknown activity relative to the different definitions of LOD: (full line) response curve of an NH_4_^+^ - selective electrode as used in this work modelled using Eq 1. (dotted line) extrapolation of responses in the cases of a_I_=0 and a_J_=0 in Eq 1. Traditional LOD defined as the cross-section of these two lines (LOD_1969_). Shaded area indicates the bias obtained in the determination of unknown activity in the region between LOD_1969_ and LOQ. Inset – illustration of detection limit defined as signal-to-noise ratio S/N=3.

## Equations for conversion

Concentrations given in this paper are reported in mg/ L or mol/ L of each analyte.

For a concentration X of nitrate, conversion from (X mg/ L) to (mol/ L) can be rewritten as;

Concentration (mol/ L) = X / 62,005

For a concentration Y of ammonium, conversion from Y (mg/ L) to (mol/ L) can be rewritten as;

Concentration (mol/ L) = Y / 18,039.

# Literature

(1) Mattinen, U.; Bobacka, J.; Lewenstam, A. Solid-Contact Reference Electrodes Based on Lipophilic Salts. *Electroanalysis* **2009**, *21* (17–18), 1955–1960. https://doi.org/10.1002/elan.200904615.

(2) Dillingham, P. W.; Alsaedi, B. S. O.; Granados-Focil, S.; Radu, A.; McGraw, C. M. Establishing Meaningful Limits of Detection for Ion-Selective Electrodes and Other Nonlinear Sensors. *ACS Sens.* **2020**, *5* (1), 250–257. https://doi.org/10.1021/acssensors.9b02133.

(3) Radu, A.; Anastasova-Ivanova, S.; Paczosa-Bator, B.; Danielewski, M.; Bobacka, J.; Lewenstam, A.; Diamond, D. Diagnostic of Functionality of Polymer Membrane–Based Ion Selective Electrodes by Impedance Spectroscopy. *Analytical Methods* **2010**, *2* (10), 1490–1498.

(4) Ainla, A.; Mousavi, M. P. S.; Tsaloglou, M.-N.; Redston, J.; Bell, J. G.; Fernández-Abedul, M. T.; Whitesides, G. M. Open-Source Potentiostat for Wireless Electrochemical Detection with Smartphones. *Analytical Chemistry* **2018**, *90* (10), 6240–6246. https://doi.org/10.1021/acs.analchem.8b00850.

(5) Fay, C.; Anastasova, S.; Slater, C.; Buda, S. T.; Shepherd, R.; Corcoran, B.; O’Connor, N. E.; Wallace, G. G.; Radu, A.; Diamond, D. Wireless Ion-Selective Electrode Autonomous Sensing System. *IEEE Sensors Journal* **2011**, *11* (10), 2374–2382.

(6) *Agriculture in the United Kingdom 2017*; DEFRA.

(7) British survey of fertiliser practice 2013 - GOV.UK https://www.gov.uk/government/statistics/british-survey-of-fertiliser-practice-2013 (accessed Dec 23, 2017).

(8) Wentworth, J. Precision Farming. **2015**.

(9) Bakker, E.; Pretsch, E.; Bühlmann, P. Selectivity of Potentiometric Ion Sensors. *Anal. Chem.* **2000**, *72* (6), 1127–1133. https://doi.org/10.1021/ac991146n.

(10) Wardak, C. Solid Contact Nitrate Ion-Selective Electrode Based on Ionic Liquid with Stable and Reproducible Potential. *Electroanalysis* **2014**, *26* (4), 864–872. https://doi.org/10.1002/elan.201300590.

(11) Pan, P. Preparation and Evaluation of a Stable Solid State Ion Selective Electrode of Polypyrrole/Electrochemically Reduced Graphene/Glassy Carbon Substrate for Soil Nitrate Sensing. *International Journal of Electrochemical Science* **2016**, *11*, 4779–4793. https://doi.org/10.20964/2016.06.7.

(12) Athavale, R.; Dinkel, C.; Wehrli, B.; Bakker, E.; Crespo, G. A.; Brand, A. Robust Solid-Contact Ion Selective Electrodes for High-Resolution In Situ Measurements in Fresh Water Systems. *Environ. Sci. Technol. Lett.* **2017**, *4* (7), 286–291. https://doi.org/10.1021/acs.estlett.7b00130.

(13) Mueller, A. V.; Hemond, H. F. Statistical Generation of Training Sets for Measuring NO3−, NH4+ and Major Ions in Natural Waters Using an Ion Selective Electrode Array. *Environ. Sci.: Processes Impacts* **2016**, *18* (5), 590–599. https://doi.org/10.1039/C6EM00043F.

(14) Paczosa-Bator, B. Effects of Type of Nanosized Carbon Black on the Performance of an All-Solid-State Potentiometric Electrode for Nitrate. *Microchim Acta* **2014**, *181* (9–10), 1093–1099. https://doi.org/10.1007/s00604-014-1216-7.

(15) Dillingham, P. W.; Radu, T.; Diamond, D.; Radu, A.; McGraw, C. M. Bayesian Methods for Ion Selective Electrodes. *Electroanalysis* **2012**, *24* (2), 316–324. https://doi.org/10.1002/elan.201100510.

(16) Durst, R. A. Ion-Selective Electrodes. 488.
